# Modeling Soluble Protein Fractionation in Feedstuffs in Equine Rations Using Crude Protein and Fiber Composition †

**DOI:** 10.3390/ani16111749

**Published:** 2026-06-05

**Authors:** Ryon W. Springer, James P. Muir, Kimberly B. Wellmann, Tryon A. Wickersham, Trinette N. Jones

**Affiliations:** 1 Department of Animal Science, Tarleton State University, Stephenville, TX 76402, USA; kwellmann@tarleton.edu (K.B.W.); tnjones@tarleton.edu (T.N.J.); 2 Texas A&M AgriLife Research, Stephenville, TX 76401, USA; jim.muir@ag.tamu.edu; 3 Department of Wildlife & Natural Resources, Tarleton State University, Stephenville, TX 76402, USA; 4 Department of Animal Science, Texas A&M University, College Station, TX 77843, USA; tryon@tamu.edu

**Keywords:** nitrogen, Akaike’s information criterion, linear regression, digestion, sustainability

## Abstract

Current National Research Council equine protein requirements are based on feedstuff crude protein (CP) content; however, protein distribution in feedstuff cell contents and cell wall affects foregut protein digestion. Neutral detergent soluble crude protein (NDSCP) is the readily digestible protein fraction available to meet equine protein requirements. Updated models of equine nutrient requirements use NDSCP to quantify pre-cecal digestible crude protein (pcdCP) to determine if protein requirements are met. Thus, our study sought to develop linear regression models to predict NDSCP and pcdCP using feedstuff CP and fiber. Data were collected from the 2023 cumulative Dairy One© Feedstuff Composition Library (Ithaca, NY, USA; accessed 2 February 2024). Feedstuffs were selected when *n* ≥ 50 within each nutrient analysis used for model development [CP, neutral detergent fiber (NDF), acid detergent fiber, acid detergent lignin]. Feedstuffs were categorized into four groups: forages, grains, grain byproducts, oilseeds and meals. Data were analyzed using multiple linear regression in R Statistical Program© (v4.5.1). Predictor variables were selected using Pearson’s correlation with models ranked using Akaike’s information criterion (AICc), AICc weights (AICcWt), and Adjusted R^2^. Significance was set at *p* ≤ 0.05. The model using CP+NDF was the highest-ranked model for both NDSCP (AICcWt = 0.95; Adjusted R^2^ = 0.9868) and pcdCP (AICcWt = 0.95; Adjusted R^2^ = 0.9867). Overall, NDSCP and pcdCP were predicted with high precision using CP and NDF; both have previously predicted CP digestibility. Overall, incorporating CP and fiber fractionation in linear models improves prediction of estimated dietary available protein.

## Simple Summary

Protein requirements for horses are based on the analysis of total nitrogen in feedstuffs, commonly known as crude protein. However, crude protein is distributed between cell contents which are available for digestion and absorption in equine small intestine, and the cell wall which is resistant to enzymatic digestion for absorption before microbial fermentation in the hindgut. Updated nutrient requirements are focused on protein found in the cell contents to improve nitrogen efficiency in the horse’s diet. However, many of the nitrogen fractions are not routinely analyzed in equine feedstuffs. Therefore, our study objective was to develop mathematical equations to predict different protein fractions in feeds commonly fed to horses. We used a large open-access database from a commercial laboratory (Dairy One© Feed Composition Library), which resulted in 74 feedstuffs across four categories that are commonly used in equine rations. Our study showed that foregut-available protein is estimated using simple crude protein and cell wall content with the respective model explaining >98% of data variability; both variables were previously used to estimate protein digestion in vivo. While our models were not validated in vivo, they provide a starting point for improving nitrogen efficiency in equine diet formulations.

## 1. Introduction

The Van Soest neutral detergent assay distinguishes between the cell wall (neutral detergent fiber: NDF) and cell contents (neutral detergent solubles). The cell wall can be partitioned further into slowly digested cell wall (acid detergent fiber: ADF) and indigestible content (acid detergent lignin: ADL) [1]. Similarly, crude protein (CP) is partitioned within plant cells between the cytoplasm (cell contents) and cell wall fractions. The Cornell Net Carbohydrate and Protein System is commonly used for ration formulation in ruminants. This system is used to estimate the partitioning of protein within the carbohydrate fractions of plant tissues with indications of bioavailability of nitrogen. Protein bound in cell walls, neutral detergent insoluble crude protein (NDICP), is resistant to chemical and enzymatic digestion in the small intestine [2]. Increases in NDICP, thus, shift protein availability from the foregut to the hindgut of the non-ruminant [3].

Previous in vivo work has shown that the majority of CP digestion, 40 to 90% of total CP digestion, occurs pre-cecally in the horse [4]. Neutral detergent soluble crude protein (NDSCP) is nitrogen that is soluble within the cytoplasm and undergoes enzymatic digestion in the stomach and small intestine of the horse; therefore, it contributes to the horse’s dietary needs [5]. A meta-analysis by Zeyner et al. [5] included diets ranging in CP from 49 to 190 g/kg DM and NDF from 195 to 732 g/kg DM to determine the relationship between NDSCP and in vivo pre-cecal digestible crude protein (pcdCP). Zeyner et al. [5] concluded that the apparent pre-cecal digestibility of NDSCP is 90% such that pcdCP can be calculated as 0.9 × NDSCP. Furthermore, Bockisch et al. [6] demonstrated a linear relationship between CP and estimated pcdCP with an 86% conversion factor for CP across a variety of feedstuffs. Increases in plasma amino acid were also associated with greater amino acid concentrations in the NDSCP fraction of different feedstuffs fed to horses, indicating the availability of amino acids within the NDSCP for pre-cecal digestion and absorption [6].

Cell wall-bound nitrogen, NDICP, can be divided further into hemicellulose-bound nitrogen (acid detergent soluble crude protein; ADSCP) and acid detergent insoluble crude protein (ADICP) [2,7]. The NDICP of feedstuffs has limited digestibility in the equine foregut but undergoes microbial fermentation in the hindgut [8]. However, ADICP is considered relatively unavailable to microbial fermentation [2]. Increases in the hemicellulose (HEMI) concentration within NDF improve post-ileal nitrogen fermentation due to greater NDF digestibility likely due to ADSCP availability to hindgut microbes and the subsequent absorption of ammonia by colonocytes [9,10]. Previous work has demonstrated a cumulative apparent fermentation of NDICP within the hindgut of 86% [8]. Jarrige and Tisserand [11] estimated the true fermentability of NDICP to be between 75% and 90%, likely from differences in HEMI and cellulose (CELL) quantities within the NDF portion of the diet. While NDICP via ADSCP may provide a nitrogen source to the hindgut microbes, there is little evidence of any hindgut free amino acids, nitrogen, or microbial cell protein serving anabolic purposes in the horse.

Previous regression models published by L’Institute National de Recherche our l’Agriculture, l’Alimentation et l’Environnement (formerly L’Institute National de Recherche; INRA) [12] show that CP digestibility in horses decreases with crude fiber, likely due to increases in fiber-bound nitrogen. Furthermore, the National Research Council (NRC) [13] reports decreased CP digestion with increases in NDF. A meta-analysis within the NRC [13] showed a strong positive correlation between available protein (AP: CP − ADICP) and total tract digestible CP, showing promise for application of protein fractionation in equine ration formulation. However, little work has been done to model CP partitioning within feedstuffs to estimate its potential availability to the horse. Therefore, our study objective was to determine if soluble protein fractions of NDSCP, pcdCP, ADSCP, and AP could be estimated using multiple linear regression equations with CP, NDF, ADF, ADL, HEMI, and CELL.

## 2. Materials and Methods

### 2.1. Data Curation

Data were collected from the 2023 cumulative Dairy One© Feedstuff Composition Library (Ithaca, NY, USA; accessed 2 February 2024). Seventy-four (*n* = 74) feedstuffs were selected based on having an n ≥ 50 samples for each protein and fiber analysis [CP, NDICP, ADICP, NDF, ADF, ADL; g/kg dry matter (DM)]. Byproducts of the alcohol fermentation process (distillers’ and brewers’ byproducts) were excluded due to microbial manipulation that could alter the nutrient composition. Nutrients not directly measured (NDSCP, ADSCP, HEMI, and CELL) were calculated using the following equations:

NDSCP (g/kg DM) = CP (g/kg DM) − NDICP (g/kg DM)(1)

ADSCP (g/kg DM) = NDICP (g/kg DM) − ADICP (g/kg DM)(2)

AP (g/kg DM) = CP (g/kg DM) − ADICP (g/kg DM)(3)

pcdCP (g/kg DM) = 0.9 × NDSCP (g/kg DM)(4)

HEMI (g/kg DM) = NDF (g/kg DM) − ADF (g/kg DM)(5)

CELL (g/kg DM) = ADF (g/kg DM) − ADL (g/kg DM)(6)

Feedstuffs were subsequently categorized into four groups: FORAGE: forages (including hays and fresh forages; *n* = 50); GRAIN: whole and processed grains (*n* = 12); GRBP: grain byproducts (*n* = 5); OSM: oilseeds and oilseed meals (*n* = 7).

### 2.2. Statistical Analysis

Data were analyzed within R Statistical Program© (v4.5.1; R Core Team, Vienna, Austria). An analysis of variance (ANOVA) was used to determine differences in protein fractionation and fiber composition among the categories of feedstuffs (mean ± SEM) with means separated using a Tukey Honest Significant Difference test. The ANOVA was performed to determine if a given predictor variable differed among the feed categories, and, therefore, was included in the model for each respective protein fraction. Correlations between the different feedstuff nutrients were determined using Pearson’s correlation. Significance was set at *p* ≤ 0.05 and tendencies determined at 0.05 < *p* ≤ 0.15.

### 2.3. Data Management and Model Development

When developing the linear regression models, predictor variables were selected for NDSCP, ADSCP, AP, and pcdCP when |*r*| > 0.2. The correlation coefficients were visualized using the *corrplot* package (v0.95) [14] with specific predictor variables eliminated for model development when *p* > 0.15 for the specific response variable. Global model predictor variables were developed based on correlation coefficients as described above as well as biological significance to the response variable. However, the global model could not contain two predictor variables with a high degree of collinearity (|*r*| > 0.7). Adjusted generalized variance inflation factors (GVIF) were determined using the *car* package (v3.1.3) [15] for the global model within each protein fraction for further prevention of multicollinearity. Variables with a GVIF > 5 were excluded from the global model.

Subsequent models for a respective response variable contained the predictor variables present in the global model. Specific feedstuffs were removed from the dataset for a given response variable when the global model was found to lack homoscedasticity as determined by the Breusch–Pagan test for non-constant error variance via the *ncvTest* function within the *car* package (v3.1.3) [15]. Beyond the initial exclusion of alcohol-production byproducts and ensiled grains and forages, palm kernel meal and sunflower meal were excluded from the modeling data for ADSCP and AP, respectively, to maintain homoscedasticity in the global model for each protein fraction. Byproducts of alcohol production and ensiled forages and grains were excluded due to microbial manipulation of fiber and protein content as well as potentially non-protein nitrogen inclusion that can alter native fiber and protein fractionation values. The Adjusted R^2^ (Adj. R^2^) and residual standard error (RSE) for each model were used to determine explanatory power of the predictor variables within a given response variable. Models were considered to have moderate and high explanatory power when Adj. R^2^ > 0.7 and Adj. R^2^ > 0.85, respectively [16]. Models within a given response variable were considered to have greater explanatory power with a smaller RSE.

### 2.4. Model Selection Using Akaike’s Information Criterion (AIC)

Multiple regression models were ranked using Akaike’s information criterion (AIC) as described by Burnham and Anderson [17]. The application of this method of model selection was previously described using in vitro digestibility data in horses [16] within the *AICcmodavg* package (v2.3.4) [18] to determine predictor variable power and rank the resulting models within a given response variable. Briefly, the AIC method uses the maximum likelihood estimation (log-likelihood: LL) for each model to measure how the data fits the model. The LL of a model is inversely related to its AIC; thus, a higher-ranked model will have a decreased AIC and increased LL. Ultimately, a decreased AIC indicates that the given model fits the response variable data better when compared to other models within the given dataset. When using AIC, the method rewards simpler models because the use of greater number of parameters is more likely to overfit a given dataset. Akaike’s information criterion (AIC) is calculated as follows:

AIC = 2*k* − 2ln(LL)

where the terms are described as:

ln = natural log;
LL = log-likelihood of the model;
*k* = (number of parameters) + 2.

The use of AIC allows for model development and selection when only a small sample size is used (*n*/*k* < 40) and model validation is not obtainable. A correction term can be used for model AIC when models developed on small samples sizes (*n*/*k* < 40) are applied to large samples sizes that results in a conservative AIC score (AICc). The AICc score was determined as:

AICc = AIC + 2*k*(*k* + 1)/(*n* − *k* + 1)

where the terms are described as:

AIC: Akaike’s information criterion score;
*k* = (number of parameters) + 2
*n* = sample size

Delta AICc (ΔAICc) was used as a metric to determine differences in evidence for the predictive power of each model within a given response variable The ΔAICc for each model is calculated by subtracting the AICc of the top-ranked model from the model in question. The thresholds for determining model strength were as follows:

ΔAICc < 2: substantial evidence for selected model;
ΔAICc < 7: moderate support for selected model;
ΔAICc < 10: low support for selected model;
ΔAICc > 10: model is very unlikely to predict the given data.

The metrics of AICc weights (AICcWt) and cumulative weights (CumWt) were used as well to determine the predictor power of a given model. The AICcWt is a ratio of the ΔAICc of the individual model compared to the whole set of candidate models, indicating the probability that the individual model is best among all the candidate models. The CumWt is the sum of the individual model and the higher-ranking models’ AICcWt [19,20]. Significance for a variable slope was set at *p* ≤ 0.05, with tendencies considered at 0.05 < *p* ≤ 0.15. Models were ranked using ΔAICc, AICcWt, and biological relevance along with goodness-of-fit slopes to determine the impact of predictor variables within a given response variable [17,18,19]. Predictor variables with increased power were visualized using the *ggplot2* package (v3.5.2) [21].

### 2.5. Model Validation

Following model development using the feed composition library, seven feedstuffs (soybean meal, whole oats, coastal Bermudagrass hay, alfalfa hay, timothy hay, Bahiagrass, mixed mostly-grass hay) were analyzed by a commercial laboratory (Dairy One©, Ithaca, NY, USA). The specific feedstuffs used in our validation were a convenience sample for other studies ongoing within our laboratory. Feedstuffs were analyzed for CP, NDICP, ADICP, NDF, ADF, and ADL. The remaining nutrients of NDSCP, pcdCP, ADSCP, AP, HEMI, and CELL were subsequently calculated using the equations presented above. The Bahiagrass sample was collected via pasture sampling across two pastures in early May 2024. The remaining feedstuffs were collected via core sampling from four bales or bags and homogenized for one laboratory analysis. The observed values from the laboratory were then compared to the expected values calculated from the top-ranked model for each protein fraction. The values were compared using Pearson’s correlation using the methods described above. Mean bias was determined for each soluble protein fraction as:

Bias (g/kg DM) = Expected (g/kg DM) − Observed (g/kg DM)(7)

Residual mean square root of prediction (RMSEP) was determined for each of the soluble protein fractions from the top-ranked model for the validation feed samples. The relative RMSEP for each soluble protein fraction was calculated to express prediction error relative to the magnitude of the observed values for the top-ranked model.

## 3. Results

### 3.1. Chemical Analyses

The chemical composition of each class of feedstuff is presented in Table 1. A total sample size of 74 feedstuffs was selected based on the set criteria. Crude protein (CP) and NDSCP were greatest in OSM compared to all other feedstuff categories (*p* < 0.001; *p* < 0.001) with no differences among the other categories (*p* ≥ 0.324; *p* ≥ 0.854). Feedstuff pcdCP was greatest in OSM (*p* < 0.001) but no difference was observed among other categories of feedstuffs (*p* ≥ 0.499). Oilseeds + oilseed meals (OSM) had the greatest ADSCP (*p* < 0.001), with FORAGE greater than GRAIN (*p* < 0.001) and both similar to GRBP (*p* = 0.903; *p* = 0.115). Total available protein (AP) was greatest in OSM (*p* < 0.001) with no differences observed among GRAIN, GRBP, and FORAGE (*p* ≥ 0.185). Fiber composition of NDF, ADF, and CELL were greatest for FORAGE (*p* ≤ 0.002; *p* ≤ 0.004; *p* ≤ 0.001, respectively) compared to all other categories. Feedstuff HEMI was similar in FORAGE and GRBP (*p* = 0.988), but both were greater than GRAIN (*p* < 0.001; *p* = 0.011, respectively). Acid detergent lignin was greatest in OSM compared to FORAGE (*p* = 0.006) which was greater than both GRAIN (*p* < 0.001) and GRBP (*p* = 0.05; Table 1).

### 3.2. Model Development

**Figure 1:**
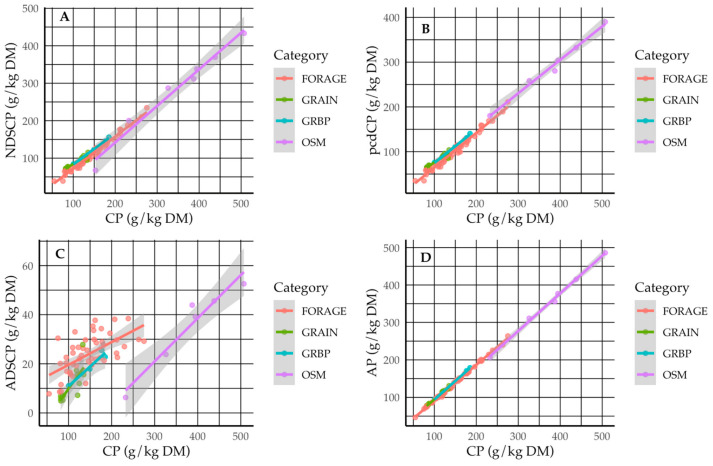
Visualization of crude protein (CP; g/kg DM) as the primary predictor variable for protein fractionation as neutral detergent soluble crude protein (NDSCP; g/kg DM; (**A**)), pre-cecal digestible crude protein (pcdCP; g/kg DM; (**B**)), acid detergent soluble crude protein (ADSCP; g/kg DM; (**C**)), and total available protein (AP; g/kg DM; (**D**)).

The correlation matrix among the different nutrients is presented in Table 2, with insignificant variables eliminated. Crude protein had strong correlations with all protein fractions (*r* ≥ 0.6531; *p* < 0.001; Figure 1). Feedstuff NDF was selected as a secondary predictor variable for NDSCP (*r* = −0.3407; *p* = 0.003) and pcdCP (*r* = −0.3407; *p* = 0.003) due to its moderate correlations and biological significance of separating the cell contents (NDSCP) and cell wall (NDICP). Other fiber analyses were not included in the model due to high collinearity with NDF (*r* ≥ 0.6432; *p* < 0.001). For ADSCP, feedstuff ADF (*r* = 0.2269; *p* = 0.054) and ADL (*r* = 0.3497; *p* = 0.002) were selected for their moderate correlations and their indications of cell wall composition, separating the hemicellulose-bound ADSCP and the cellulose-bound ADICP. Total available protein included HEMI (*r* = −0.3747; *p* = 0.001) and ADL (*r* = 0.2206; *p* = 0.061) due to the increased correlation of HEMI with AP compared to NDF (*r* = −0.2819; *p* = 0.016) and the lack of correlation between HEMI and ADL (*r* = 0.1507; *p* = 0.203).

### 3.3. Model Selection

The predictor variables for the NDSCP global model had GVIF ≤ 1.96. The model of CP+NDF was the highest-ranked model for predicting NDSCP (AICcWt = 0.95; Adj. R^2^ = 0.9868; RSE = 8.54). When accounting for categorical differences (CP+NDF+CAT), there was a decrease in explanatory power (Adj. R^2^ = 0.9864; RSE = 8.65). The second-ranked model (CP+NDF+CAT) had only moderate predictor power (AICcWt = 0.05) and moderate support for the model (ΔAICc = 6.13). No other models were within the threshold of ΔAICc ≤ 10.0 as CumWt reached 1.00 with the first two models (Table 3 and Table 4).

The pcdCP global model contained predictor variables with GVIF ≤ 1.96. Pre-cecal digestible crude protein was best predicted using CP+NDF (AICcWt = 0.95; Adj. R^2^ = 0.9867; RSE = 7.70). Categorical adjustments (CP+NDF+CAT) decreased explanatory power (Adj. R^2^ = 0.9863; RSE = 7.81) compared to the top-ranked model and only had moderate predictor power (AICcWt = 0.05) and moderate support for the model (ΔAICc = 5.92). No other models were within the threshold of ΔAICc ≤ 10.0, as CumWt reached 1.00 with the first two models (Table 5 and Table 6).

All predictor variables for the ADSCP global model had GVIF ≤ 2.37. The top-ranked model for ADSCP was the global model (CP+ADF+ADL+CAT; AICcWt = 0.90). However, moderate evidence was present for CP+ADL+CAT (ΔAICc = 5.92) and CP+ADF+ADL (ΔAICc = 6.40). Regarding explanatory power, CP+ADF+ADL+CAT had moderate explanatory power (Adj. R^2^ = 0.7169; RSE = 5.50), similar to CP+ADL+CAT (Adj. R^2^ = 0.6869; RSE = 5.78) and CP+ADF+ADL (Adj. R^2^ = 0.6732; RSE = 5.91). The AICc.Wt was 0.99 when accounting for the three top-ranked models; therefore, the remaining models were considered to have little predictor power (Table 7 and Table 8).

Total soluble protein was best predicted using the global model (CP+HEMI+ADL+CAT; AICcWt = 0.81) with all predictor variables having GVIF < 1.74. However, there was strong moderate evidence for CP+HEMI+ADL (ΔAICc = 2.96; AICcWt = 0.18). There was low support for the third-ranked model of CP+ADL (AICcWt = 0.01; ΔAICc = 8.91). While the top-ranked model had a slightly greater explanatory power (Adj. R^2^ = 0.9993; RSE = 1.985), the second-ranked (Adj. R^2^ = 0.9992; RSE = 2.086) and third-ranked models (Adj. R^2^ = 0.9992; RSE = 2.195) also had high model fits. All other models were unlikely to predict the given data (ΔAICc > 10.0; Table 9 and Table 10).

### 3.4. Model Validation

The expected values from our secondary feedstuff analysis for NDSCP (*r* = 0.9968; *p* < 0.001), pcdCP (*r* = 0.9968; *p* < 0.001), and AP (*r* = 0.9991; *p* < 0.001) obtained from the highest-ranked model for each analysis were highly correlated with the observed values. However, predicted ADSCP values were not correlated (*r* = 0.2325; *p* = 0.424) with the observed values. Mean prediction bias was low for all response variables (ADSCP = 2.26, NDSCP = −2.58, pcdCP = −2.11, and AP = 1.46 g/kg DM), while RMSEP values were 14.45, 10.56, 9.57, and 6.14 g/kg DM, respectively. The relative RMSEP was relatively small for NDSCP (6.34%), pcdCP (6.38%), and AP (3.16%), but was very high for ADSCP (56.20%; Table 11).

## 4. Discussion

Three primary systems for providing the nutrient requirements of horses include the NRC [13], INRA [12], and Gesellschaft für Ernährungsphysiologie (GfE) [22]. While the NRC [13] still uses CP as the base requirement for average maintenance in the horse (1.26 g/kg BW/d), INRA [12] uses horse digestible crude protein (MADC) system (2.8 g/kg BW^0.75^/d), and the GfE [22] system is based on pcdCP (3.0 g/kg BW^0.75^/d). The NRC assumes a total tract apparent digestibility of CP to be 73–83% for alfalfa hay, 57–64% for coastal Bermudagrass hay, and 67–74% for other forages [13]. While the European nutrient requirement systems attempt to improve protein efficiency in the horse, the NRC [13] only uses CP without fractionation considerations, the GfE [22] requires additional laboratory analyses for calculation of estimated pcdCP, and INRA [12] uses crude fiber, a biologically irrelevant analysis, for estimating protein digestion without understanding actual protein fractionation in the feedstuff. Our study attempts to better understand protein fractionation in relation to fiber content in feedstuffs to later improve relation between the three prominent protein systems. However, it should be noted that horses require amino acids in the diet, not simply CP, to meet daily nutrient requirements for tissue development and repair [12,13,22]. While our proposed models present ways to better predict protein fractionation for application within the current nutrient requirement framework, we did not determine the quality of protein, and therefore, more work is warranted for modeling amino acid content and fractionation within the protein fractions [6].

From our models, pcdCP and NDSCP decrease with NDF composition, decreasing CP available for enzymatic digestion in the stomach and small intestine. This mechanism is plausible as there is an increase in NDF from alfalfa to grass hays [13]. The estimates obtained for pcdCP from our models for alfalfa hay, coastal Bermudagrass hay, and timothy hay were 150.7 g/kg DM, 96.1 g/kg DM, and 63.2 g/kg DM, respectively. The estimated pcdCP model outputs convert to 73.5, 66.3, and 62.6% CP, respectively, which align with the NRC [13] values. From previous literature, hindgut microbes utilize fiber-bound nitrogen in some capacity [8], provided that there is enough nitrogen from the overflow of NDSCP from the foregut [5], or that they use urea recycled back to the colon to maintain nitrogen supply for microbial metabolism [10]. Bockisch et al. [6] provided a linear regression for predicting pcdCP model using CP as a predictor variable with a high correlation coefficient (*r* = 0.967). However, our top-ranked model (CP+NDF) showed a lesser variable slope for CP to predict pcdCP (0.762) but a similar slope for NDSCP (0.847) compared to Bockisch et al. [5]. Farley et al. [23] showed that when fiber was held constant (estimated at approximately 300 g NDF/kg DM), pre-cecal digestibility of CP was 0.722, similar to the coefficient for CP (0.762) within our top-ranked model for pcdCP. Neutral detergent fiber increases while CP decreases with plant maturity [24,25]. As NDF increases, greater CP is bound within the cell wall with plant maturity and therefore decreases NDSCP available for digestion in the equine small intestine [24]. In swine, NDF decreases ileal nitrogen digestibility while increasing fecal output of nitrogen, indicating an increase in fiber-bound nitrogen with increasing NDF content [26]. The increase in NDF likely decreases NDSCP and subsequently pcdCP by binding nitrogen within the cell wall as the plant matures. Bocksich et al. [6] showed that aNDFom (NDF exclusive of acid detergent insoluble ash) had a moderate negative correlation (r = −0.573) with pre-cecal digestibility of CP (% CP), having a variable slope of −0.039. The current study showed that NDF had a low negative correlation with pcdCP and NDSCP (*r* = −0.3407) with similar variable slopes (−0.031; −0.036, respectively) compared to Bockisch et al. [6]. While the simple regression model by Bockisch et al. [6] only used CP for estimating pcdCP, there were differences in the feedstuff category NDF content in the current study. Therefore, NDF improved the predictor power of the model which increased the likelihood that CP+NDF will best predict future data for both NDSCP and pcdCP [17]. However, NDSCP and pcdCP are not actual analyses and are determined arithmetically from the analysis of CP and NDICP. Therefore, there is an innate analytical error within these protein fractions. Furthermore, more work is warranted on the impact of feed processing on application of our proposed models, specifically for predicting NDSCP and pcdCP. Previous work by Pisch et al. [27] showed that steaming hay at 97 °C for 1 h increased NDICP and NDF but reduced NDSCP and pcdCP and likely AP. Limiting amino acids, specifically lysine, threonine, and methionine, had reduced estimated pre-cecal digestibility as they became bound within the cell wall by increasing the concentration of Maillard reaction products within the hay [27]. Therefore, caution is warranted when applying our proposed models to feedstuffs at high risk of Maillard reaction productions until further model validation is performed.

Models developed in our study failed to adequately predict ADSCP concentrations in sampled feedstuffs. The observed and predicted values from the top-ranked model had a low correlation (*r* = 0.2325; *p* = 0.424). The current study showed that NDF had a low correlation to ADSCP (*r* = 0.2204) with greater correlations for ADL (*r* = 0.3497) and ADF (*r* = 0.2269). Neither ADF nor ADL improved the model predictability which indicated that variables absent from the dataset are likely more powerful predictor variables [17]. Forage ADSCP increases with alfalfa maturity. However, the same trend was not observed for bromegrass or either endophyte-infected or -free tall fescue as NDF, ADF, and ADL all increased with maturity [24]. Another study showed no changes in ADSCP among maturity stages of alfalfa hay, but the study also did not observe changes in NDF or ADF as there was only a 14-day difference among cutting maturities [25]. Work by Gustavsson and Martinsson [28] showed that NDF, ADF, and CELL increased with plant maturity while HEMI and ADL did not follow the same linear line. Furthermore, the authors noted a decrease in ADSCP with plant growth, but the composition of HEMI, ADSCP, and ADL within NDF changed in response to environmental factors beyond plant maturity [28]. Plant HEMI is a heterogenous mixture of xylans, xylaglucans, mannans, and glucomannans with their composition within the cell wall highly variable. Due to its structure, the enzymes required for HEMI production, which account for a large portion of the ADSCP, are also heterogenous [29]. As this study did not measure HEMI composition, this confounding factor may account for the variability in protein composition within the enzymatic structure of the cell wall.

The fractions of NDSCP and AP are CP-derived outcomes [2,13], with pcdCP being a simple conversion from NDSCP [5], while ADSCP is not. The top-ranked model for predicting ADSCP failed the validation step of our study, as the predicted versus observed ADSCP values were not correlated (*r* = 0.2325; *p* = 0.424). As CP accounted for a large amount of variation in each of the respective models, as shown in Figure 1, the inability to predict ADSCP from our models is likely due to physiological variables that are not captured by the analyses used in our models. Additionally, non-linear model development may be required when predicting fiber-bound nitrogen. From a nutritional perspective, ADSCP has not been extensively researched within equine nutrition. While INRA [12] does acknowledge the microbial need for protein-based nitrogen, there is little data that assesses the contribution of ADSCP to the equine diet. Previous work by Glade [8] demonstrated that NDICP had approximately 43% fermentability in the hindgut of the horse, which was likely ADSCP due to the limited availability of ADICP to microbes [2,3]. However, the correlation of ADSCP digestibility to other dietary nutrients was not investigated and may not be easily estimated. Therefore, it may be more useful to predict NDSCP and AP to arithmetically calculate ADSCP. However, the arithmetic method was not validated in our study and, therefore, may incur the error associated with using two predictive models.

The AP fraction that our study investigated was calculated as NDSCP + ADSCP or CP − ADICP. The MADC system by INRA [12] would likely be most comparable to AP. Our study used the predictor variables of CP, HEMI, and ADL to explain the current data. The slopes of our study within the two top-ranked models ranged from 0.971 to 0.967 for CP, 0.009 to 0.011 for HEMI, and −0.134 to −0.138 for ADL. Compared to the MADC system, CP was much greater in this study compared to INRA [12] (0.725 versus 0.894), but the MADC horse models only used crude fiber as a cell wall variable, while our study focused on cell wall nutritive value. The combination of HEMI and ADL outputs within our study would likely be comparable to the crude fiber contributions to the MADC models. According to Van Soest et al. [2], ADICP can be considered unavailable to the gastrointestinal microbial populations. Total AP does not distinguish the differences in CP efficiency between the foregut, estimated at 86% of CP [6], and the hindgut, estimated at 43% of NDICP [8]. However, it may provide an indication of total available CP within the diet. Pagan [30] found that the true digestibility of CP was 86%, while other researchers have demonstrated a range of 87 to 96% true total tract digestibility [9,31]. When converting our estimated AP values from our top-ranked model using nutrient values within the NRC for alfalfa hay (186.1 g/kg DM), coastal bermudagrass hay (133.8 g/kg DM), and timothy hay (87.7 g/kg DM), to percent-CP basis, their respective AP values (92.6, 92.2, and 89.5%, respectively) fall within the ranges of previous estimated true CP digestibility [9,30,31]. A previous meta-analysis by the NRC [13] showed a strong correlation between digestible CP and AP (R^2^ = 0.94). However, the NRC [13] only plotted digestible CP to predict AP and not vice versa; subsequently, a conversion factor of AP to digestible CP was not presented. However, more work is warranted regarding the application of our models, specifically the AP model, to feedstuffs with greater quantities of ADICP. Sniffen et al. [7] listed specific feedstuffs with large amounts of ADICP as hay-crop silages, dehydrated alfalfa meal, citrus pulp, corn distillers grains, and brewers dried grains. Within our models, we excluded the ensiled grains and forages and alcohol-production byproducts because of potential microbial manipulation of the protein and fiber profiles. We also did not include citrus pulp because it failed to meet the minimum threshold of *n* ≥ 50 for each nutrient analysis while dehydrated alfalfa meal was not included in the library database. Therefore, it may be more useful to develop separate models for estimating protein fractionation for feedstuffs with greater quantities of ADICP, or to analyze the protein fractions directly if using those specific ingredients listed above. However, AP may be useful to predict the total digestible protein within the equine diet without accounting for differences in nitrogen efficiency in different segments of the equine gastrointestinal tract.

## 5. Conclusions

The three main nutrient requirement systems differ in terms of their recommendations, including CP, pcdCP, and MADC. However, there has been no direct way of estimating protein fractionation in feedstuffs through linear regression models using CP and fiber composition. Our objective was to develop multiple linear regression models to predict feedstuff NDSCP, pcdCP, ADSCP, and AP using AICc model selection. This study showed that NDSCP, pcdCP, and AP could be predicted with high precision using CP and fiber composition. However, the models developed did not predict ADSCP fractions of analyzed feedstuffs, likely due to environmental factors or other nutrients that were not accounted for within the data set. The models developed in this study for NDSCP, pcdCP, and AP provide a reference point to better determine protein digestion in the small intestine and nitrogen fermentation in the hindgut and further correspond to the protein requirement standards for horses among the NRC [12], GfE [21], and INRA [11] systems. However, in vivo digestibility trials are warranted to validate the implications of our predictive modeling for dietary formulation.

## Figures and Tables

**Table 1:** Chemical composition (g/kg DM) of various forages (FORAGE), grains and processed grains (GRAIN), grain byproducts (GRBP), and oilseeds and their meals (OSM).

Mean ± SEM ^1^ (Min–Max)	FORAGE ^2^ (g/kg DM) ^1^	GRAIN ^3^ (g/kg DM) ^1^	GRBP ^4^ (g/kg DM) ^1^	OSM ^5^ (g/kg DM) ^1^
n	50	12	5	7
CP	146.11 ± 7.11 ^b^ (54.8–275.2)	113.89 ± 12.88 ^b^ (81.7–135.2)	100.5 ± 15.29 ^b^ (100.5–256.8)	348.36 ± 46.01 ^a^ (133.0–507.1)
NDSCP	111.54 ± 6.18 ^b^ (38.7–234.5)	97.83 ± 11.42 ^b^ (68.3–115.4)	120.70 ± 13.88 ^b^ (84.4–168.9)	286.71 ± 45.50 ^a^ (67.5–433.3)
pcdCP	100.30 ± 5.57 ^b^ (34.8–211.1)	79.21 ± 4.21 ^b^ (61.5–103.9)	114.48 ± 14.25 ^b^ (76.0–152.0)	258.04 ± 40.99 ^a^ (60.8–390.0)
ADSCP	23.84 ± 1.09 ^b^ (7.8–38.4)	10.41 ± 1.56 ^c^ (4.9–17.3)	21.02 ± 2.98 ^bc^ (11.1–57.6)	38.76 ± 6.89 ^a^ (6.3–60.0)
AP	135.38 ± 6.78 ^b^ (46.5–263.7)	99.05 ± 6.09 ^b^ (75.2–131.0)	146.53 ± 18.87 ^b^ (95.5–226.5)	325.47 ± 46.52 ^a^ (122.5–485.9)
NDF	536.13 ± 13.74 ^a^ (177.4–726.6)	143.54 ± 16.38 ^c^ (86.0–270.5)	360.80 ± 81.11 ^b^ (169.0–505.5)	357.07 ± 73.21 ^b^ (86.9–642.6)
HEMI	183.20 ± 8.51 ^a^ (53.4–308.8)	75.85 ± 10.02 ^c^ (22.9–135.1)	174.42 ± 30.53 ^ab^ (99.5–255.3)	105.59 ± 24.04 ^bc^ (13.1–219.4)
ADF	352.92 ± 7.62 ^a^ (124.0–500.1)	67.68 ± 8.71 ^c^ (35.9–135.4)	186.38 ± 70.89 ^b^ (58.3–250.2)	251.49 ± 50.95 ^b^ (73.8–463.3)
CELL	298.29 ± 7.19 ^a^ (105.8–427.2)	51.03 ± 7.53 ^c^ (23.5–104.3)	153.74 ± 7.16 ^b^ (44.0–182.8)	174.49 ± 35.64 ^b^ (60.8–437.6)
ADL	54.64 ± 2.04 ^b^ (18.2–96.4)	16.66 ± 1.59 ^c^ (11.4–31.1)	32.64 ± 5.56 ^c^ (14.3–67.4)	79.00 ± 16.23 ^a^ (13.0–123.4)

^a,b,c^ Means within row lacking common superscripts differ (*p* ≤ 0.05). ^1^ Results presented as mean ± standard error of the mean (mean ± SEM). ^2^ Forages. ^3^ Grains and processed grains. ^4^ Grain byproducts. ^5^ Oilseeds and oilseed meals. Abbreviations: CP: crude protein; NDSCP: neutral detergent soluble crude protein; pcdCP: pre-cecal digestible crude protein; ADSCP: acid detergent soluble crude protein; AP: total available protein; NDF: neutral detergent fiber; ADF: acid detergent fiber; ADL: acid detergent lignin; HEMI: hemicellulose; CELL: cellulose.

**Table 2:** Correlations among feedstuff crude protein, neutral detergent soluble crude protein, pre-cecal digestible crude protein, acid detergent soluble crude protein, total soluble protein, and fiber fractions.

Item	NDSCP	pcdCP	ADSCP	AP	NDF	ADF	ADL	HEMI	CELL
CP	0.9901 (<0.001)	0.9901 (<0.001)	0.6531 (<0.001)	0.9988 (<0.001)	−0.2643 (0.024)	−0.1692 (0.152)	0.2582 (0.027)	−0.3730 (0.001)	−0.2454 (0.036)
NDSCP	---------	---------	0.5458 (<0.001)	0.9932 (<0.001)	−0.3407 (0.003)	−0.2449 (0.037)	0.1875 (0.112)	−0.4319 (<0.001)	−0.3154 (0.007)
pcdCP	---------	---------	0.5458 (<0.001)	0.9932 (<0.001)	−0.3407 (0.003)	−0.2449 (0.037)	0.1875 (0.112)	−0.4319 (<0.001)	−0.3154 (0.007)
ADSCP			---------	0.6397 (<0.001)	0.2204 (0.061)	0.2269 (0.054)	0.3497 (0.002)	0.1544 (0.192)	0.1803 (0.127)
AP				---------	−0.2819 (0.016)	−0.1931 (0.102)	0.2206 (0.061)	−0.3747 (0.001)	−0.2642 (0.024)
NDF					---------	0.9543 (<0.001)	0.6432 (<0.001)	0.8384 (<0.001)	0.9629 (<0.001)
ADF						---------	0.6432 (<0.001)	0.6372 (<0.001)	0.9865 (<0.001)
ADL							---------	0.1507 (0.203)	0.5092 (<0.001)
HEMI								---------	0.6840 (<0.001)
CELL									---------

Upper row: Pearson’s correlation coefficient. Lower row in parentheses: *p*-value. Abbreviations: CP: crude protein; NDSCP: neutral detergent soluble crude protein; pcdCP: pre-cecal digestible crude protein; ADSCP: acid detergent soluble crude protein; AP: total available protein; NDF: neutral detergent fiber; ADF: acid detergent fiber; ADL: acid detergent lignin; HEMI: hemicellulose; CELL: cellulose.

**Table 3:** Estimation of neutral detergent soluble crude protein from feedstuff crude protein and neutral detergent fiber composition.

Model	k	AICc	ΔAICc	AICcWt	CumWt	LL
CP+NDF	4	532.87	0.00	0.95	0.95	−262.15
CP+NDF+CAT	7	538.79	5.92	0.05	1.00	−261.55
CP+CAT	6	543.37	10.50	0.00	1.00	−265.06
CP	3	563.36	30.49	0.00	1.00	−278.51
NULL	2	850.73	317.86	0.00	1.00	−423.28

Abbreviations: CP: crude protein (g/kg DM); NDF: neutral detergent fiber (g/kg DM); CAT: category of feedstuff; k: (number of parameters) + 2; AICc: Akaike’s information criterion with correction factor; ΔAICc: delta AICc from top-ranked model; AICcWt; individual AICc weight; CumWt: cumulative weight of models; LL: log-likelihood.

**Table 4:** Linear and multiple regression models predicting feedstuff neutral detergent soluble crude protein from crude protein and neutral detergent fiber composition.

Model	Intercept	CP	NDF	GRAIN	GRBP	OSM	RSE	Adj. R^2^
CP+NDF	6.677	0.847	−0.036	-----	-----	-----	8.54	0.9868
CP+NDF+CAT	3.375 ^a^	0.863	−0.033	0.996 ^a^	0.872 ^a^	−0.526 ^a^	8.65	0.9864
CP+CAT	−20.786	0.906	-----	15.471	6.595 ^b^	−7.998 ^b^	11.07	0.9851
CP	−12.619	0.868	-----	-----	-----	-----	10.57	0.9797

Abbreviations: CP: crude protein (g/kg DM); NDF: neutral detergent fiber (g/kg DM); CAT: category of feedstuff; GRAIN: grains and processed grains; GRBP: grain byproducts; OSM: oilseeds and oilseed meals; RSE: residual standard error; ^a^ *p* > 0.15; ^b^ 0.05 < *p* ≤ 0.15.

**Table 5:** Estimation of pre-cecal digestible crude protein from feedstuff crude protein and neutral detergent fiber.

Model	k	AICc	ΔAICc	AICcWt	CumWt	LL
CP+NDF	4	510.63	0.00	0.95	0.95	−251.02
CP+NDF+CAT	7	517.76	6.13	0.05	1.00	−250.52
CP+CAT	6	524.54	13.92	0.00	1.00	−255.64
CP	3	538.85	28.22	0.00	1.00	−266.25
Null	2	823.52	312.90	0.00	1.00	−266.25

Abbreviations: CP: crude protein (g/kg DM); NDF: neutral detergent fiber (g/kg DM); CAT: category of feedstuff; k: (number of parameters) + 2; AICc: Akaike’s information criterion with correction factor; ΔAICc: delta AICc from top-ranked model; AICcWt; individual AICc weight; CumWt: cumulative weight of models; LL: log-likelihood.

**Table 6:** Linear and multiple regression models predicting feedstuff pre-cecal digestible crude protein from feedstuff crude protein and neutral detergent fiber.

Model	Intercept	CP	NDF	GRAIN	GRBP	OSM	RSE	Adj. R^2^
CP+NDF	5.654 ^a^	0.762	−0.031				7.70	0.9867
CP+NDF+CAT	4.829 ^b^	0.771	−0.032	−0.586 ^b^	1.033 ^b^	−3.881 ^b^	7.81	0.9863
CP+CAT	−18.210	0.812		12.229	8.560 ^a^	−6.509 ^b^	8.32	0.9884
CP	−11.189	0.779					9.41	0.9801

Abbreviations: CP: crude protein (g/kg DM); NDF: neutral detergent fiber (g/kg DM); CAT: category of feedstuff; GRAIN: grains and processed grains; GRBP: grain byproducts; OSM: oilseeds and oilseed meals; RSE: residual standard error; ^a^ *p* > 0.15; ^b^ 0.05 < *p* ≤ 0.15.

**Table 7:** Estimation of acid detergent soluble crude protein from feedstuff crude protein, acid detergent fiber, and acid detergent lignin composition.

Model	k	AICc	ΔAICc	AICcWt	CumWt	LL
CP+ADF+ADL+CAT	8	466.92	0.00	0.90	0.90	−224.34
CP+ADL+CAT	7	472.84	5.92	0.05	0.95	−228.56
CP+ADF+ADL	5	473.32	6.40	0.04	0.99	−231.21
CP+ADF+CAT	7	475.65	8.73	0.01	1.00	−229.96
CP+ADF	4	486.95	20.03	0.00	1.00	−239.18
CP+ADL	4	505.07	38.15	0.00	1.00	−248.24
Null	2	551.32	84.40	0.00	1.00	−273.57

Abbreviations: CP: crude protein (g/kg DM); ADF: acid detergent fiber (g/kg DM); ADL: acid detergent lignin (g/kg DM); CAT: category of feedstuff; k: (number of parameters) + 2; AICc: Akaike’s information criterion with correction factor; ΔAICc: delta AICc from top-ranked model; AICcWt; individual AICc weight; CumWt: cumulative weight of models; LL: log-likelihood.

**Table 8:** Linear and multiple regression models predicting feedstuff acid detergent soluble crude protein from crude protein, acid detergent fiber, and acid detergent lignin composition.

Model	Intercept	CP	ADF	ADL	GRAIN	GRBP	OSM	RSE	Adj. R^2^
CP+ADF+ADL+CAT	−0.995 ^a^	0.134	0.038	−0.150	−3.970 ^a^	−0.175 ^a^	−12.791	5.50	0.7169
CP+ADL+CAT	12.724	0.109	-----	−0.087	−13.242	−5.045 ^b^	−12.663	5.78	0.6869
CP+ADF+ADL	−1.517 ^a^	0.113	0.053	−0.192	-----	-----	-----	5.91	0.6731
CP+ADF+CAT	−0.269 ^a^	0.125	0.017 ^a^	-----	−4.694 ^a^	−0.426 ^a^	−15.907	5.90	0.6746
CP+ADF	−0.382 ^a^	0.093	0.028	-----	-----	-----	-----	6.54	0.5991
CP+ADL	7.514	0.083	-----	0.032 ^a^	-----	-----	-----	7.41	0.4862

Abbreviations: CP: crude protein (g/kg DM); ADF: acid detergent fiber (g/kg DM); ADL: acid detergent lignin (g/kg DM); CAT: category of feedstuff; GRAIN: grains and processed grains; GRBP: grain byproducts; OSM: oilseeds and oilseed meals; RSE: residual standard error; ^a^ *p* > 0.15; ^b^ 0.05 < *p* ≤ 0.15.

**Table 9:** Estimation of available protein from feedstuff crude protein, hemicellulose, and acid detergent lignin composition.

Model	k	AICc	ΔAICc	AICcWt	CumWt	LL
CP+HEMI+ADL+CAT	8	309.77	0.00	0.81	0.81	−145.72
CP+HEMI +ADL	5	312.73	2.96	0.18	0.99	−150.90
CP+ADL	4	318.68	8.91	0.01	1.00	−155.04
CP+CAT	6	375.19	65.42	0.00	1.00	−180.94
CP	3	396.37	86.60	0.00	1.00	−195.00
CP+HEMI	4	398.43	88.66	0.00	1.00	−194.91
Null	2	818.58	508.81	0.00	1.00	−407.20

Abbreviations: CP: crude protein (g/kg DM); HEMI: hemicellulose (g/kg DM); ADL: acid detergent lignin (g/kg DM); CAT: category of feedstuff; k: (number of parameters) + 2; AICc: Akaike’s information criterion with correction factor; ΔAICc: delta AICc from top-ranked model; AICcWt; individual AICc weight; CumWt: cumulative weight of models; LL: log-likelihood.

**Table 10:** Linear and multiple regression models predicting available protein from feedstuff crude protein, hemicellulose, and acid detergent lignin composition.

Model	Intercept	CP	HEMI	ADL	GRAIN	GRBP	OSM	RSE	Adj. R^2^
CP+HEMI+ADL+CAT	−0.527 ^a^	0.971	0.009 ^b^	−0.138	−0.614 ^a^	1.182 ^b^	−2.811	1.985	0.9993
CP+HEMI+ADL	−0.240 ^a^	0.967	0.011	−0.143				2.086	0.9992
CP+ADL	1.750	0.963		−0.134				2.195	0.9992
CP+CAT	−7.497	0.978			3.921	4.483	−7.543	3.209	0.9982
CP	−3.751	0.956						3.826	0.9974
CP+HEMI	−3.142 ^a^	0.955	−0.003 ^a^					3.849	0.9974

Abbreviations: CP: crude protein (g/kg DM); HEMI: hemicellulose (g/kg DM); ADL: acid detergent lignin (g/kg DM); CAT: category of feedstuff; GRAIN: grains and processed grains; GRBP: grain byproducts; OSM: oilseeds and oilseed meals; RSE: residual standard error; ^a^ *p* > 0.15; ^b^ 0.05 < *p* ≤ 0.15.

**Table 11:** Diagnostic statistics for expected values of six analyzed feedstuffs used to validate the top-ranked model of each soluble protein fraction.

Protein Fraction	Observed Min–Max (g/kg DM)	Mean Observed (g/kg DM)	Mean Expected (g/kg DM)	Pearson’s Correlation (*p*-Value)	RMSEP (g/kg DM)	Bias (g/kg DM)	Relative RMSEP (%)
NDSCP	78.0–433.0	167.1	164.8	0.9968 (<0.001)	10.6	−2.3	6.34
pcdCP	70.2–389.7	150.4	148.3	0.9968 (<0.001)	9.6	−2.1	6.38
ADSCP	13.0–44.0	25.8	28.0	0.2325 (0.424)	14.5	2.2	56.20
AP	93.0–463.0	192.8	194.3	0.9991 (<0.001)	6.1	1.5	3.16

Abbreviations: NDSCP, neutral detergent soluble crude protein; pcdCP, pre-cecal digestible crude protein; ADSCP, acid detergent soluble crude protein; AP, total available protein; RMSEP, Root mean square error of prediction.

## Data Availability

The original data presented in the study are openly available at https://dairyone.com/services/forage-laboratory-services/feed-composition-library/ (accessed on 2 February 2024).

## Abbreviations

The following abbreviations are used in this manuscript:

AIC	Akaike’s information criterion
AICc	AIC with correction factor for small n/k ratio
ΔAICc	Change in AICc from top-ranked model
AICcWt	Percent of future data where model is top-ranked
ADF	Acid detergent fiber
ADL	Acid detergent lignin
ADICP	Acid detergent insoluble crude protein
Adj. R^2^	Adjusted R^2^
ADSCP	Acid detergent soluble crude protein
ANOVA	Analysis of variance
AP	Total available protein
CELL	Cellulose
CP	Crude protein
CumWt	Cumulative AICcWt of top-ranked models
FORAGE	Forages
GfE	Gesellschaft für Ernährungsphysiologie
GRAIN	Grains and processed grains
GRBP	Grain byproducts
HEMI	Hemicellulose
INRA	L’Institute National de Recherche our l’Agriculture
LL	Log-likelihood
NDF	Neutral detergent fiber
NDICP	Neutral detergent insoluble crude protein
NDSCP	Neutral detergent soluble crude protein
NRC	National Research Council
OSM	Oilseeds and oilseed meals
pcdCP	Pre-cecal digestible crude protein
RMSEP	Root mean square error of prediction
RSE	Residual standard error
